# The nature and origins of political polarization over science

**DOI:** 10.1177/0963662521989193

**Published:** 2021-02-17

**Authors:** Roderik Rekker

**Affiliations:** University of Gothenburg, Sweden

**Keywords:** ideology, motivated reasoning, partisanship, polarization, science rejection

## Abstract

People have a tendency to disregard information that contradicts their partisan or ideological identity. This inclination can become especially striking when citizens reject notions that scientists would consider “facts” in the light of overwhelming scientific evidence and consensus. The resulting polarization over science has reached alarming levels in recent years. This theoretical review conceptualizes political polarization over science and argues that it is driven by two interrelated processes. Through *psychological science rejection*, people can implicitly disregard scientific facts that are inconsistent with their political identity. Alternatively, citizens can engage in *ideological science rejection* by adhering to a political ideology that explicitly contests science. This contestation can in turn be subdivided into four levels of generalization: An ideology can dispute either specific scientific claims, distinct research fields, science in general, or the entire political system and elite. By proposing this interdisciplinary framework, this article aims to integrate insights from various disciplines.

## 1. Introduction

Whereas the vast majority (84%) of American citizens who identify as a Democrat accept the scientific consensus that climate change is caused by human activity, less than half (43%) of Republicans share this position (Dunlap et al., 2016). This partisan divide becomes even wider in combination with ideology. Nearly all liberal Democrats (94%) believe that climate change is a major threat, but only a small minority of conservative Republicans agrees (19%; Kennedy and Hefferon, 2019). Moreover, this divide has widened sharply since the beginning of the twenty-first century (Dunlap et al., 2016). The American dispute over climate change therefore illustrates a broader phenomenon of increasing partisan and ideological polarization over science that can also be observed in other countries (Tranter, 2011; Whitmarsh, 2011), for other scientific claims (Hamilton, 2015), and for trust in science as a whole (Gauchat, 2012).

There are at least two reasons why such polarization could be normatively problematic. First, it is commonly believed that a democratic debate over policy requires at least some basic agreement on facts (Haas, 2018). As Delli Carpini and Keeter (1996: 8, 11) put it, facts are “the currency of citizenship” that “prevent debates from becoming disconnected from the material conditions they attempt to address.” Second, the idea that scientific inquiry is the basis for informed policymaking lies at the core of modern society (Lasswell, 1951). This pivotal role of science may however be threatened when it is no longer recognized as an impartial and trustworthy authority.

The increasing political polarization over science has therefore received ample attention, but the scholarly literature is dispersed over a wide variety of academic disciplines such as psychology, sociology, communication, and political science. Each field is characterized by its own distinct problem definition, terminology, explanatory models, and methodological approach. Although each field has undoubtedly provided highly valuable insights, it has therefore become a complex puzzle how all these accounts relate to each other and which approach best explains a particular case. This theoretical review aims to address this puzzle by providing an interdisciplinary framework that conceptualizes what political polarization over science is and how it may be explained.

The first section of this article will conceptualize political polarization over science as “partisan or ideological alignment over the authority of science or a scientific consensus.” The second and third section will subsequently distinguish two interrelated but conceptually distinct explanatory models. Through *psychological science rejection*, people can implicitly disregard scientific facts that are inconsistent with their political identity. Alternatively, citizens can engage in *ideological science rejection* by adhering to a political ideology that explicitly contests science. The fourth and fifth section will then respectively examine how psychological and ideological science rejection may be distinguished and how both accounts may be used to explain why polarization over science has increased over time.

## 2. Conceptualizing science polarization

Polarization over science is a multifaceted phenomenon that has been approached from different angles because it can be conceptualized in various ways. In an attempt to bring these approaches together, this section will first conceptualize what political polarization over science is and what it is not. Throughout this article, political polarization over science will also be abbreviated as *science polarization*. Providing a definition of science polarization first requires a definition of what political polarization is and second a conceptualization of when science should be considered its object.

The first definition relates to a theoretical debate on whether political polarization should be defined in terms of *divergence* or *alignment* (Lelkes, 2016). Following the divergence account, polarization can be defined as the “extent to which opinions on issues are opposed in relation to some theoretical maximum” as well as the “increase in such opposition over time” (DiMaggio et al., 1996). Based on the alignment perspective, political polarization can contrarily be viewed as the degree to which citizens’ positions on a given issue are defined by their partisan or ideological identity (Abramowitz and Saunders, 2008). This article will draw from the alignment account because the phenomenon that needs to be explained is not so much an increasing overall variation in attitudes toward science, but rather why these views have become increasingly connected to citizens’ political identity.

**Figure 1.**
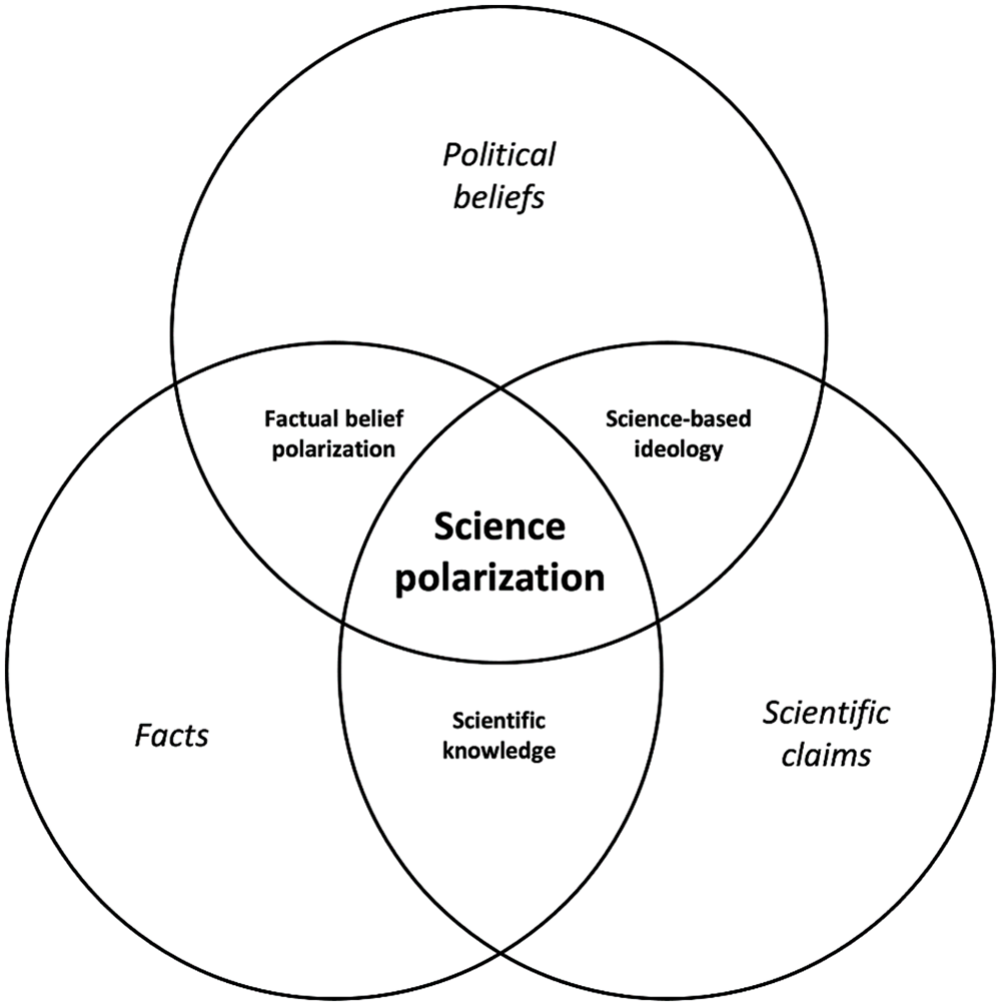
Conceptualizing the object of science polarization as the intersection between scientific claims, facts, and political beliefs.

To define the *object of science polarization*, it is useful to reflect on the intersections between three phenomena as depicted in Figure 1: scientific claims, facts, and political beliefs. First, the intersection between scientific claims and facts can be viewed as *scientific knowledge*. An example is the notion that water molecules are composed of two hydrogen atoms and one oxygen atom. This is a scientific claim because the public learns about it from scientific inquiry. The question why this statement can also be considered a “fact” is more complex and can be answered from two ontological perspectives. From a realist ontology, it can be argued that the scientific method is the best available source of objective knowledge that corresponds with an external reality (Leplin, 1984). Because the composition of water molecules is supported by strong scientific evidence, it may therefore be considered a fact from this perspective because it is “true.” From a pragmatist viewpoint, it can additionally be reasoned that the functioning of liberal democracy requires that citizens accept information as fact when it comes from sources that function as an impartial source of information. For example, democratic societies have given various public agencies the task and recourses to provide factual information on matters such as the size of unemployment or the amount of air pollution. Regardless of whether such institutions speak the “truth” in its realist meaning, societal cohesion and democratic government may require that their authority is accepted and hence that the information they provide has the status of “facts” in the public debate and policymaking. From this perspective, it can be argued that it is normatively desirable when society accepts the composition of water molecules as a fact because there is consensus among those who have been given the task and resources to provide factual information on molecular physics.

The second intersection is the one between scientific claims and political beliefs. An example is the economic idea that budgetary austerity is a necessity to regain long-term economic growth during an economic crisis. This notion is a scientific claim because it has been argued by economists who base their claims on scientific inquiry. However, the necessity of austerity cannot be considered a fact because there is no scientific consensus on this matter. Based on equally rigorous scientific inquiry, a sizable number of economists have argued that austerity policies do more harm than good (Blanchard and Leigh, 2013). Crucially, how citizens perceive the economic effect of austerity is also part of their political belief system due to its direct relevance for contested budgetary measures. The acceptance or rejection of austerity policies can therefore be conceptualized as *science-based ideology* because these political beliefs are substantiated by scientific claims. This case should therefore not be seen as an example of science polarization because it involves *contestation based on science* rather than *contestation of science*. Both sides acknowledge the authority of science and use it to substantiate their policy proposals and to contest those of their rivals.

The third intersection can be found between facts and political beliefs. An example is the statement that the US government spends more money on health care than on the military. Drawing from the logic outlined earlier, this information should be considered a fact because it comes from an institution (i.e., the Congressional Budget Office) that has been given the task and the recourses to provide factual information on this issue. Nonetheless, Democrats and Republicans are strongly divided in their factual perceptions of this issue which indicates that it is also part of citizens’ political belief systems. Democrats are more likely than Republicans to hold the misperception that the US government instead spends more money on defense than on health care (Lee et al., 2020). Such misperceptions moreover cannot be attributed simply to a lack of awareness because they often persist even after citizens have been correctly informed (Nyhan and Reifler, 2010). These partisan and ideological divides over facts can be referred to as *factual belief polarization* (Lee et al., 2020).

The fourth and final intersection is the one where scientific claims, facts, and political beliefs all come together. An example is the notion that global warming is caused by human activity. Like the composition of water molecules, this statement can be considered scientific knowledge because it is subject to scholarly consensus (Cook et al., 2013). Like the distribution of government spending, climate change is also an object of factual belief polarization because citizens are divided along partisan and ideological lines in their factual perceptions. Like the need for budgetary austerity, it is furthermore an object of science-based ideology. While some political actors have used climate science to substantiate pleas for a reduction of CO_2_ emissions, others have attempted to find or develop a counterargument to prevent such policies (Brulle, 2014). Because those counterarguments lack support from within the scientific community, those efforts have however taken the form of a *contestation of science* rather than a *contestation based on science*. In other words, this is a situation in which a science-based ideology (i.e., a political agenda to reduce CO_2_ emissions) is challenged by another ideology that is not based on science, but rather on fake experts or pseudoscience. Whereas both sides used scientific evidence in the debate over budgetary austerity, the discussion over climate change has therefore become one in which the authority of science itself is questioned. In such instances, science becomes politicized and citizens become polarized in their acceptance or rejection of its authority. For precisely this reason, the intersection between scientific claims, facts, and political beliefs can be viewed as the object of science polarization.

While the contestation of a scientific consensus indirectly results in political polarization over the authority of science, this polarization can also manifest itself more directly. Even outside the context of politicized scientific claims, citizens are also divided along partisan and ideological lines over the authority and trustworthiness of science as a whole. For example, American citizens who identify as liberal have more trust in science than conservatives and this gap has widened considerably over the years (Gauchat, 2012). Like polarization over a scientific consensus, such partisan and ideological divides over the authority of science as a whole can also be viewed as a manifestation of science polarization.

Based on the aforementioned conceptualization of political polarization and this demarcation of when science should be considered its object, science polarization can now be defined as “partisan or ideological alignment over the authority of science or a scientific consensus.” Although it is beyond the scope of this article to conceptualize when we should speak of “scientific consensus,” this definition can be applied in conjunction with existing work on this question (e.g., Shwed and Bearman, 2010). This definition of science polarization is also in line with existing conceptualizations of related phenomena that also are based on the notion of scientific consensus. For example, Bolsen and Druckman (2015: 65) defined “politicization of science” as “when an actor emphasizes the inherent uncertainty of science to cast doubt on the existence of scientific consensus.” Likewise, Nyhan and Reifler (2010: 305) defined “misperceptions” as “cases in which people’s beliefs about factual matters are not supported by clear evidence and expert opinion.” Besides divisions over climate change and trust in science, other manifestations that fall under this definition of science polarization include partisan and ideological contestation over whether vaccines cause autism, whether condoms reduce the risk of AIDS, or whether the human species has evolved over time. Science polarization can furthermore be seen as a specific manifestation of factual belief polarization and may be distinguished from other forms of polarization such as ideological polarization and affective polarization. The utility of this definition lies in its ability to bring together various lines of research that have approached the same phenomenon from a different angle. The following sections will therefore aim to integrate explanations for science polarization from a variety of research fields.

## 3. Psychological science rejection

Explaining science polarization revolves around the question why citizens’ partisan or ideological identity would lead them to reject science or scientific claims. For many years, psychologists have sought to answer this question by examining psychological processes at the level of individual citizens. Psychology has furthermore approached science rejection mainly as a specific manifestation of factual belief polarization (see Figure 1). From a psychological viewpoint, the question why citizens would reject a scientific consensus is not fundamentally different from the question why they would reject any other type of fact. More specifically, psychologists have therefore examined why people have a psychological tendency to disregard factual information that is incongruent with their political identity.

In answering this question, psychologists have proposed two competing sets of explanations (Nisbet et al., 2015): the intrinsic thesis and the contextual thesis. The *intrinsic thesis* holds that there is something unique about the psychology of conservatives. Dating back to Adorno et al.’s (1950) classic work “The Authoritarian Personality,” an extensive literature has argued that conservatives are characterized by distinct personality traits such as “dogmatism,” “intolerance of uncertainty,” and “need for closure” (Jost et al., 2003). The intrinsic thesis argues that these traits make conservatives much more likely than liberals to disregard information that challenges their ideological worldview (Kruglanski, 2004). In other words, this account attributes science polarization to an inherently anti-scientific personality of citizens on one, but not the other, side of the political spectrum. In support of this explanation, numerous studies have demonstrated cognitive differences between liberals and conservatives in which the latter were more likely to avoid dissonant information or dissonant-arousing tasks (e.g., Nam et al., 2013; Shook and Fazio, 2009).

The intrinsic thesis has however been challenged by two types of criticism. A soft critique argues that subtle cognitive differences between liberals and conservatives should not be overstated and that they should not be seen as the primary cause of factual belief polarization (Nisbet et al., 2015). A hard critique of the intrinsic thesis goes a step further by challenging the entire notion that conservatives are more likely than liberals to engage in motivated reasoning (Kahan, 2012, 2016). This criticism has questioned the validity of the reasoning measures that conservatives score lower on, as well as the claim that such measures are associated with the kind of motivated reasoning that causes factual belief polarization. This critique has furthermore questioned the internal validity of experiments supporting the intrinsic thesis by arguing that their findings can also be explained by more rational forms of belief updating (Kahan, 2016). This criticism is supported by studies that instead rely on more stringent measures and designs. Providing strong evidence against the intrinsic thesis, a meta-analysis of such studies revealed that liberals and conservatives are equally biased in their cognition of counterattitudinal information (Ditto et al., 2019).

Based on such findings, the literature overall seems to be shifting away from the intrinsic thesis as the main explanation for factual belief polarization. The alternative to the intrinsic thesis comes from a set of explanations that is known as the *contextual thesis*. This account argues that factual belief polarization is the result of a dynamic interplay between institutional and psychological factors, in which the politicization of factual claims interacts with citizens’ partisan and ideological identity (Nisbet et al., 2015). From this perspective, factual belief polarization can be explained by a process of *politically motivated reasoning* that is driven by people’s psychological need to form beliefs that maintain their status in an affinity group (Cohen, 2003). This process is also referred to as *identity-protective cognition*. The strongest evidence for this interpretation comes from experiments that revealed how subjects’ processing of information was determined by an interaction between their political identity and an experimentally manipulated identity congruence of stimulus material (Kahan, 2016). For example, one of these studies experimentally manipulated the identity relevance of anthropogenic climate change by altering its implications for contested environmental policies. The results revealed that rightist subjects were more likely to credit evidence on anthropogenic climate change after being exposed to information on how CO_2_ regulations were rendered unnecessary by a new technology, whereas leftist subjects credited the evidence less in this condition (Kahan et al., 2015).

In sum, psychology has provided an explanation for science polarization by examining why individual citizens tend to reject identity-incongruent information. This article will use the term *psychological science rejection* to refer to the combined insights of psychological explanations in general and the idea of identity-protective cognition in particular. Specifically, psychological science rejection can be defined as “the process through which individual citizens implicitly disregard scientific claims that are inconsistent with their group identity.” Conservatives may for example have an implicit tendency to avoid, ignore, or distrust information from climate scientists because endorsing the idea of anthropogenic climate change would threaten their conservative identity and their status in the affinity group of conservatives.

## 4. Ideological science rejection

As discussed in the previous section, psychologists have examined why individual citizens tend to reject identity-incongruent scientific claims. In this approach, the content of political ideologies and the politicization of scientific claims are treated as exogeneous variables. This leaves open the question why scientific claims would be incompatible with partisan or ideological identities in the first place. Answering this question requires a shift away from the level of individual citizens, toward the aggregate and institutional level of political ideologies, parties, and interest groups. This approach has been taken mainly by scholars in sociology, political science, and communication. Drawing from the conceptualization of science polarization in Figure 1, this perspective approaches the phenomenon from the angle of *science-based ideology*.

The question why an ideology would contest science is closely connected to the question why it would endorse it. Philosophers have typically distinguished science and ideology as the difference between “what is” and “what ought to be” or between epistemology and ethics (Nutini, 1971). This distinction has however always been more complicated in practice. Most political ideologies make strong statements about how the world is, as well as about how it should be. As a result, political ideologies have often incorporated scientific ideas. Social democracy has for example been influenced by Keynesian economics, while contemporary economic conservatism has been inspired by new classical economics (Adams, 1993; Van Horn and Mirowski, 2009). Likewise, many political parties have founded their own think tanks with the explicit aim to ground their ideology in academic literature (McGann, 2018). Although few would question the merits of such *science-based ideology*, this inevitably means that ideologies also use their own scientific foundation to contest the scientific claims of their rivals. Whereas the latter case can still be viewed as an instance of science-based ideology, this is no longer the case for ideologies that use claims from fake experts or pseudoscience to challenge the scientific basis of rivaling political agendas.

**Figure 2.**
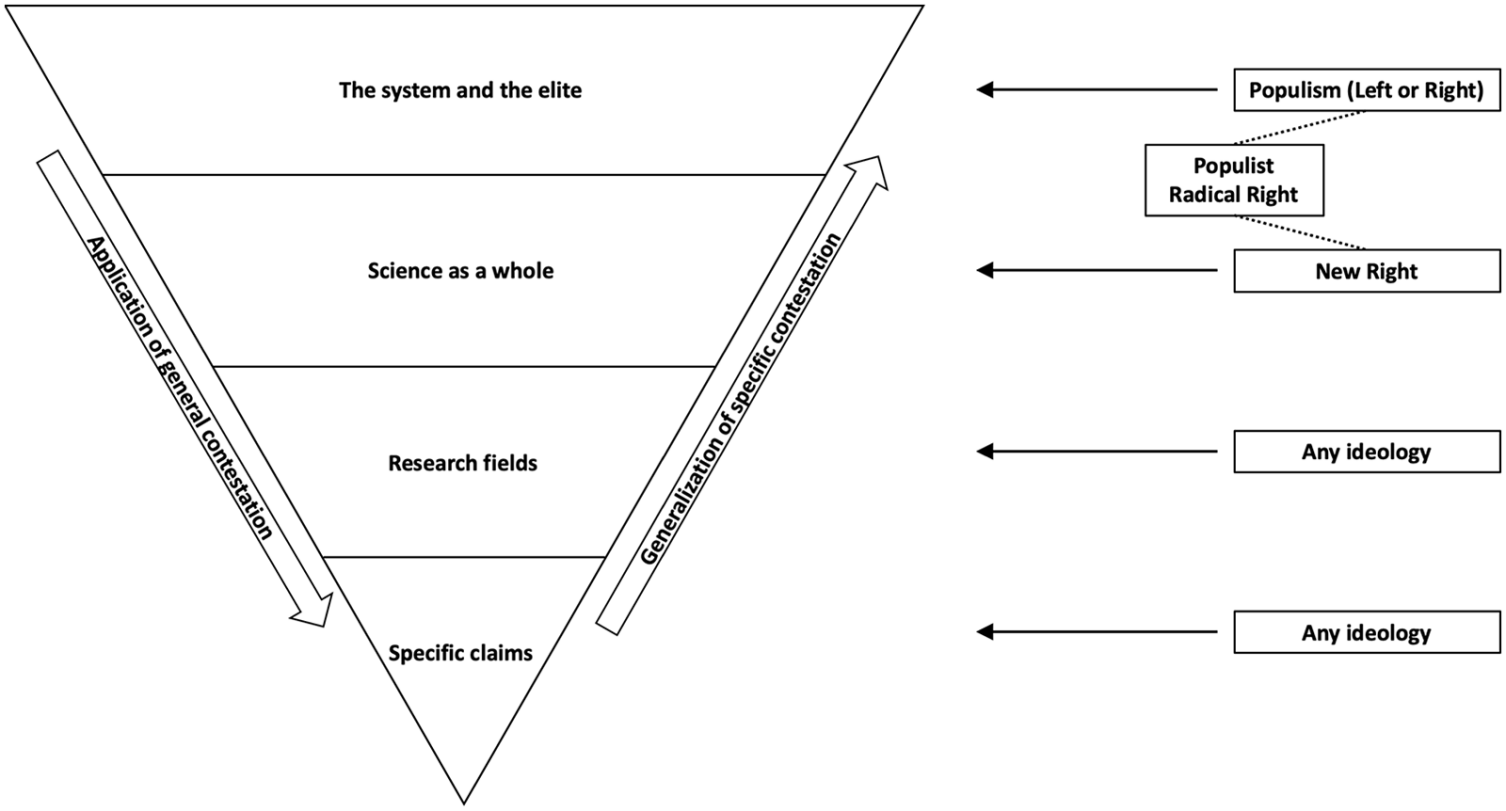
The levels and processes of ideological science rejection.

Such ideological opposition to science can exist on four different levels ranging from *specific contestation* to *general contestation* as depicted in Figure 2. First, an ideology can dispute *specific scientific claims*. The political Right has for example traditionally been skeptical about the Keynesian idea that governments can alleviate economic recessions by borrowing money for public works, whereas the Left has commonly questioned claims from new classical economics about the benefits of tax cuts and international free trade (Adams, 1993; Berman and Milanes-Reyes, 2013). Although ideas from economics and the social sciences have therefore always been subject to politicization, this has not always been the case for the natural sciences. For a long time, physical and engineering sciences were depoliticized as a source of technological progress that benefits everyone (Mooney, 2005). Since about the 1970s, there has however been a growing concern about the impact of such technological advances on the environment and public health. As a result, a new type of regulatory science has emerged to examine this impact and to guide environmental policies (Jasanoff, 1990; Yearley, 1994). This new role has contributed to an increasing politicization of the natural sciences because, much like economics, it had now become the basis for controversial policies (Mooney, 2005). For example, political movements and industries that oppose CO_2_ regulations have sought to develop their own counterargument. This has resulted in a variety of think tanks that are known as the “Climate Change Counter Movement” (CCCM). By supporting such organizations, the American conservative movement has developed an elaborate ideological opposition to the notion of anthropogenic climate change. For example, research reveals that the CCCM receives over US$900 million in annual donations from conservative foundations (Brulle, 2014) and that it has had a substantial impact on public opinion and policy in the United States (Dunlap and Jacques, 2013; McCright and Dunlap, 2010).

A second level of ideological opposition arises from the fact that some controversial ideas and technologies are intimately associated with the academic disciplines that generated them. In such instances, entire *research fields* can become politicized. For example, the political Right has traditionally been critical about the entire endeavor of regulatory science. According to the *anti-reflexivity thesis*, this should be seen as an effort to defend the industrial capitalist order against scientific claims that the current economic system causes ecological and public health problems (Dunlap, 2014; McCright and Dunlap, 2010). Christian conservative groups have similarly raised fundamental moral objections against stem cell research and some environmentalist organizations have opposed the entire principle of genetic engineering (Nisbet, 2004; Reisner, 2001). Such ideological opposition to research fields is also reflected in public opinion (McCright et al., 2013): Conservatives tend to report less trust than liberals in scientists who examine the impact of economic production on the environment and public health (i.e., impact science), whereas liberals reversely express less trust than conservatives in scientists who develop innovations for economic production (i.e., production science).

At a third level of ideological contestation, political ideologies can also differ in their views on the role of *science as a whole*. Whereas any ideology may occasionally question specific claims or research fields, a more general opposition to science can be observed mainly on the right side of the political spectrum. For example, Mooney (2005) argued in his book “The Republican War on Science” that science polarization in the United States has been driven by the emergence of the “New Right” since the 1970s. This opposition may be explained by a combination of ideological and political motivations. First, the conservative ideology has for many centuries been more critical than other ideologies about the central role of science in modern societies. Conservative philosophers like Edmund Burke have for example questioned the modernist idea that science should be used as a driver of rapid and rational societal change (Elofson and Woods, 1998). Moreover, conservatives have traditionally been uncomfortable with the notion that science should have a monopoly on truth claims. Many conservatives instead view science as just one source of knowledge among others such as religion, tradition, or “common sense” (Bowler, 2010; Gauchat, 2011). Mooney (2005) furthermore reasoned that the New Right’s mobilization against science may be explained by its origins as an electoral alliance of the Religious Right and transnational corporations. Whereas Christian conservatives have a religious motivation to contest science over issues like Darwinian evolution and stem cell research, corporations have an economic interest in challenging science over regulatory policies. According to Mooney (2005), this wide array of political and ideological motivations has accumulated in a more generalized opposition by the New Right against science as a whole. This opposition has yielded a large number of think tanks that the New Right has used to develop its own alternative source of information (Nash, 2014; Oreskes and Conway, 2011). Such alternative truth claims could subsequently be spread by the New Right’s extensive media empire, which includes book publishing houses, radio, cable television, and Internet sites (Blee and Creasap, 2010; Davis and Owen, 1998). The New Right has thus challenged the authority of science by developing an extensive apparatus for the conception and proliferation of alternative worldviews. This account of elite-driven science polarization in the United States is supported by research that shows how over-time increases in this polarization coincide with electoral and ideological breakthroughs of the New Right (Gauchat, 2012).

A fourth and final level of opposition arises from the fact that universities are often public institutions and that scientists are inevitably part of a highly educated cultural elite. Ideological contestation of science can therefore be part of an even broader opposition against *the system and the elite*. Most noticeably, the ideology of populism claims that politics is about a fundamental divide between a corrupted elite and a homogeneous people (Mudde, 2007). Populism can hence be viewed as a “thin ideology” that can supplement any “host ideology” on either the left or the right side of the political spectrum. Research shows that the anti-elite rhetoric of populist parties has fueled distrust among their voters (Rooduijn et al., 2016). As a result, citizens’ trust in public institutions has become increasingly associated with their party preference (Van der Meer, 2017). Since citizens’ trust in various public institutions is strongly intercorrelated, this general polarization over trust in public institutions has most likely contributed to a similar polarization over trust in science. Further evidence for the role of populism in science polarization comes from research that shows how citizens on both extreme ends of the left-right continuum are more likely to believe in simple political solutions and, as a result, to endorse the kind of conspiracy theories that often underlie science rejection (Lewandowsky et al., 2013; Van Prooijen et al., 2015). Indicating that this association constitutes a causal effect of populism, experimental research shows that anti-elite rhetoric can activate anti-intellectual attitudes that are strongly associated with science rejection (Merkley, 2020; Motta, 2018). In this regard, the “Populist Radical Right” (PRR) seems particularly relevant. This ideology is the most important manifestation of populism in Europe and North America, while it can also be seen as an ideological exponent of the aforementioned New Right (Oudenampsen, 2018). Unsurprisingly, PRR voters in Western Europe are indeed far more likely than voters of all other parties to hold misperceptions, to distrust science, and to reject the notion of anthropogenic climate change (Huber, 2020; Van Kessel et al., 2021; Van Vliet, 2019).

Although ideological contestation of science seems to be initiated mostly by institutional actors such as political parties and think tanks, individual citizens may be influenced because they take cues from such elites in determining their own views. An extensive body of research shows how people form their attitudes in accordance with their partisan and ideological identity (e.g., Campbell et al., 1960). Cues from elites generally play an important role in this process (Bakker et al., 2019) and research shows that this is also the case for attitudes on climate change (Merkley and Stecula, 2020). Citizens who identify as conservative may therefore adopt negative views about science when it is contested by conservative institutions such as parties and news media (Hmielowski et al., 2014). Although ideological contestation of science can exist on four different levels as discussed earlier, this contestation may easily travel from one level to another. On one hand, numerous studies have revealed how citizens use more general levels of trust as a heuristic to form more specific attitudes (e.g., Brossard and Nisbet, 2007; Hmielowski et al., 2014). Citizens who distrust science as a whole may therefore also be more likely to reject specific scientific claims (i.e., *application of general contestation*). Reversely, research (Nisbet et al., 2015) has also shown how citizens who reject science on specific issues may resultingly become distrustful of science as a whole (i.e., *generalization of specific contestation*).

In sum, this explanation for science polarization describes an elite-driven process in which public opinion is shaped by institutional actors that contest and politicize science. This article will refer to this set of explanations with the term *ideological science rejection*. Specifically, ideological science rejection can be defined as “the process through which citizens or institutional actors conceive, proliferate, or embrace an ideology that explicitly contests science.” Conservatives may for example reject anthropogenic climate change because they take cues from conservative politicians and media who spread an elaborate narrative about why climate science should not be trusted.

## 5. Distinguishing psychological and ideological science rejection

The previous sections discussed psychological science rejection (PSR) and ideological science rejection (ISR), but the crucial question how these two accounts relate to each other has not yet been addressed. Some may argue that PSR and ISR should not be seen as distinct mechanisms, but rather as two facets of the same phenomenon. In favor of this interpretation is the fact that PSR cannot explain science polarization without some assumptions about the politicization of science by elites. While PSR can explain why citizens reject scientific claims that are incongruent with their political identity, it cannot explain why some scientific claims would be incompatible with certain political identities in the first place. ISR can reversely explain how elites politicize some scientific claims, but it requires psychological assumptions to explain how this would result in science polarization among the general public. As such, PSR and ISR are importantly interrelated and interdependent.

This argument notwithstanding, this article argues that PSR and ISR should nonetheless be seen as conceptually distinct explanations for science polarization (see Table 1). On one hand, PSR can exist with only very minimal ideological cues. Citizens can implicitly reject identity-incongruent facts even in the absence of any explicit elite cues, as long as they experience some discrepancy between a scientific claim and their worldview. On the other hand, ISR requires only very minimal psychological assumptions to explain science polarization. The mere idea that citizens are more likely to take cues from politicians or news media from their own ideological camp can already explain why elite-driven efforts to contest science can cause science polarization among the general public. Even though PSR and ISR are not mutually exclusive and importantly interrelated, this indicates that they nonetheless constitute conceptually distinct processes. Importantly, this implies that PSR may best explain some manifestations of science rejection, whereas ISR may best describe others.

**Table 1.** Overview of the theoretical distinction between psychological and ideological science rejection.

	Psychological science rejection	Ideological science rejection
Definition	Implicitly disregarding science	Explicitly contesting science
Mostly studied by	Psychology	Sociology, political science, communication
Larger phenomenon	Biased cognition of counterattitudinal information	Interconnectedness of science and political ideology
Related terminology	Politically motivated reasoning, identity protection	Politicization of science, organized science denial, misinformation
Who are rejecting?	Individual citizens	Citizens or institutional actors
What is rejected?	Specific claims (but contestation may generalize, see Figure 2)	Specific claims, research fields, science in general, elite/system
How is it rejected?	Rejecting identity-incongruent claims from scientists	Embracing identity-congruent ideology from political elites
Which side is rejecting?	Depends on issue	Depends on issue/field (specific), New Right/populists (general)
Over-time increase due to	Increasing affective polarization, increasing education	New ideologies, politicization of trust, polarization/relativism in media, increasing education

There are at least two ways to translate the distinction between PSR and ISR to empirical research. A first distinction is one *between* different research paradigms. Whereas PSR can be examined primarily with experimental studies on how individual citizens process information (e.g., Kahan, 2016), ISR may be studied with a variety of designs and units of analysis. How scientific claims have become politicized may for example be studied by using (automated) content analyses on news content (e.g,. Schmid-Petri, 2017) or statements from politicians (e.g., Brown and Sovacool, 2017). Longitudinal surveys can in turn be used to examine if the politicization of science by elites co-occurs with shifts in public opinion among citizens with different partisan and ideological orientations (e.g., Gauchat, 2012). Experimental designs can finally determine when and from whom citizens take cues on scientific issues (e.g., Porter et al., 2019).

A second operational distinction between PSR and ISR can be made *within* empirical studies. For example, experimental studies could simultaneously examine how citizens’ political identity leads them to reject claims from scientists (i.e., PSR) or to accept counter-scientific statements from politicians (i.e., ISR), as well as the interaction between both processes. Likewise, studies can assess to what extent respondents’ rejection of science is explicit. Because PSR is mostly implicit (Cohen, 2003), it may be taken as a sign of ISR when respondents can explicitly motivate their rejection of science with elaborate counter-scientific narratives or conspiracy theories. Empirical research may furthermore disentangle PSR and ISR by examining mediating and moderating processes. PSR may for example be moderated by the strength of citizens’ political identity (Kahan, 2016), whereas ISR may be strengthened by citizens’ susceptibility to conspiracy theories (Van Prooijen et al., 2015) or their use of ideological news media (Iyengar and Hahn, 2009).

## 6. Explaining the increase in science polarization

This article started with the observation that science polarization has increased sharply over time. Once again, the psychological and ideological paradigm provide different explanations for this development. PSR may have increased over time as a result of increasing levels of *affective polarization*. Particularly in the United States, citizens express increasing levels of sympathy toward partisan or ideological in-groups and increasing antagonism toward out-groups (Iyengar et al., 2012; Reiljan, 2019). When citizens become more emotionally invested in their political identity, they may also become more likely to engage in motivated reasoning to protect this identity (Garrett et al., 2019).

Drawing from the perspective of ISR, the over-time increase in science polarization may contrarily be explained by the rise of new ideologies that contest science (Mooney, 2005). This account is supported by studies that show how over-time increases in science polarization correspond with the breakthrough of the New Right in the United States (Gauchat, 2012) and the Populist Radical Right in Western Europe (Van Vliet, 2019). Related to the rise of populism, increases in ISR may also be driven by the increasing politicization of political trust. Citizens’ trust in science may have become polarized because their trust in public institutions has more generally become interconnected with their partisan and ideological preferences (Van der Meer, 2017). The intensifying debate over far-reaching environmental policies may also have contributed to an increasing polarization over science, for example in the case of climate science after the Paris Agreement.

Various contributions from communication science can also be placed under the umbrella of ISR. Such accounts have related increases in science polarization to over-time changes in the media environment. Since the 1990s, the media landscape has become increasingly polarized and fragmented (Van Aelst et al., 2017). Most importantly, there seems to have been a growing supply of and demand for ideological news (Arceneaux and Johnson, 2013) in both online and offline media. Such ideological news media may have greatly facilitated the proliferation of anti-scientific ideology, as well as the polarization of citizens’ worldviews more generally (Davis and Dunaway, 2016; Tsfati et al., 2014). Research for example shows that the use of conservative media decreases citizens’ trust in science, which in turn decreases their certainty that global warming is happening, whereas the use of non-conservative media has the opposite effect (Hmielowski et al., 2014). Another aspect of the media environment that may have facilitated science polarization is *relativism* in the coverage of scientific issues. Some news media treat science as just one opinion among others, for example by giving equal attention to scientists and science skeptics (Van Aelst et al., 2017). Research shows that exposure to such journalistic “false balance” can distort citizens’ perceptions of expert opinion on issues like vaccines or climate change (Dixon and Clarke, 2013; Koehler, 2016).

Ironically, increases in science polarization may furthermore be explained by the fact that citizens’ educational attainment has increased over time. Increases in PSR could be explained by increasing education because those citizens who have the greatest capacity to understand scientific information are often most likely to engage in politically motivated reasoning (Kahan et al., 2017). Increases in ISR may also be explained by education because the most politically sophisticated citizens have the strongest tendency to bring their attitudes in line with their political identity by for example taking cues from elites (Bakker et al., 2019; Converse, 1964; Zaller, 1992). As a result, partisan and ideological divides over science are often widest among highly educated citizens (Hamilton, 2011, 2016; McCright and Dunlap, 2011).

## 7. Conclusion

Partisan and ideological polarization over science have increased to alarming levels over the past decades. The extensive scholarly literature on this phenomenon is however dispersed over a wide variety of academic disciplines with distinct approaches. This theoretical review aimed to integrate these accounts by providing an interdisciplinary framework that conceptualizes what science polarization is and how it may be explained. To this end, this article distinguished two types of explanatory mechanisms: psychological and ideological science rejection. Even though both mechanisms are importantly interconnected, this article argued that they constitute conceptually distinct explanations for science polarization. Although this article focused specifically on *political* polarization over science, the models that were discussed can also be applied to many non-political forms of science rejection. Psychological science rejection can for example also be driven by non-political group identities, while ideological science rejection may also involve the explicit rejection of science based on non-political belief systems such as conspiracy theories.

The framework that was outlined in this article may serve as a starting point to both separate and integrate insights from various disciplines. Any instance of science polarization may be explained by either psychological or ideological science rejection, or a combination of both. Research should therefore not attribute a particular manifestation to either of the two mechanisms without considering this distinction. For example, the fact that conservatives are more likely to distrust science is sometimes taken as evidence that they have a stronger psychological tendency to engage in motivated reasoning, while this difference could also be explained by ideological science rejection or by other confounding factors. Indeed, this article argued that liberals and conservatives are equally predisposed to engage in psychological science rejection or to explicitly reject scientific claims and research fields, but that some types of conservatives are more likely to reject science in general as part of their ideology.

A key challenge for future research is therefore to distinguish psychological and ideological science rejection more explicitly and to examine what mechanism offers the best explanation for each specific case of science polarization such as a particular issue, country, or group of citizens. This article provided some suggestions for how this distinction could be implemented empirically. Such research may also inform communication strategies for combating science polarization. Psychological science rejection may for example be countered by communicating science to skeptics with a rhetoric that is more consistent with their political identity (Feinberg and Willer, 2013; Nan and Madden, 2014). Contrarily, ideological science rejection may for example be combatted by exposing the sources of anti-scientific narratives and their interests (MacFarlane et al., 2020). Another direction for future research is to determine whether science polarization takes place on a more specific or a more general level (see Figure 2). Some citizens may for example reject the notion of anthropogenic climate due to a more general distrust of science, whereas others may only oppose this specific idea. Likewise, a distrust of science among voters of Populist Radical Right parties may or may not be reducible to a more general lack of confidence in public institutions among this group. Future research may also examine the role of increasing affective polarization in psychological science rejection, as well as the role of the increasing availability of ideological news in ideological science rejection. Finally, there is an urgent need for more research outside the American context. Science polarization also seems on the rise in Western Europe (Van Vliet, 2019), but hardly any studies have been conducted in this context. Particularly the role of Populist Radical Right parties and their electorates should be examined further in these countries.
